# Twenty years of telemedicine in chronic disease management – an evidence synthesis

**DOI:** 10.1258/jtt.2012.120219

**Published:** 2012-06

**Authors:** Richard Wootton

**Affiliations:** Norwegian Centre for Integrated Care and Telemedicine, Tromsø, Norway

## Abstract

A literature review was conducted to obtain a high-level view of the value of telemedicine in the management of five common chronic diseases (asthma, COPD, diabetes, heart failure, hypertension). A total of 141 randomised controlled trials (RCTs) was identified, in which 148 telemedicine interventions of various kinds had been tested in a total of 37,695 patients. The value of each intervention was categorised in terms of the outcomes specified by the investigators in that trial, i.e. no attempt was made to extract a common outcome from all studies, as would be required for a conventional meta-analysis. Summarizing the value of these interventions shows, first, that most studies have reported positive effects (*n* = 108), and almost none have reported negative effects (*n* = 2). This suggests publication bias. Second, there were no significant differences between the chronic diseases, i.e. telemedicine seems equally effective (or ineffective) in the diseases studied. Third, most studies have been relatively short-term (median duration 6 months). It seems unlikely that in a chronic disease, any intervention can have much effect unless applied for a long period. Finally, there have been very few studies of cost-effectiveness. Thus the evidence base for the value of telemedicine in managing chronic diseases is on the whole weak and contradictory.

## Introduction

Chronic illnesses, such as asthma, COPD, diabetes, heart failure and hypertension represent a significant burden of disease. Burden of disease is measured in Disability-Adjusted Life Years (DALYs), which reflect years of life lost from premature death and years of life lived in less than full health. In high-income countries, asthma, COPD and diabetes represent 11.1 million DALYs or 7% of the total DALYs.^1^ As well as their significance from the perspective of those affected, chronic diseases also impose huge costs on the health care systems responsible for managing them. In the US, the direct health care costs for patients with asthma, diabetes, heart disease and hypertension were $52.1 billion in 1996.^2^

Does telemedicine have a role in the management of chronic diseases? Before considering this question, it is worth thinking about where telemedicine would fit into the disease management process. Most of us would imagine, a priori, that closer involvement of health care staff with a patient who has one or more chronic diseases would reduce morbidity and perhaps mortality. There is some evidence, for example, that use of nurse case managers (combined with a patient education programme) is efficacious.^3^ Use of case managers is one aspect of providing “integrated care”, a fashionable term with a rather elastic definition. Integrated care is commonly thought of as a process that seeks to achieve seamless and continuous care, tailored to the individual patient's needs, and based on a holistic view of the patient. There are several synonyms, such as disease management, care management, managed care and coordinated care. Integrated care programmes seem to have positive effects on the quality of care, although the widely varying definitions and components may lead to inappropriate conclusions being drawn.^4^

How has telemedicine been used to support integrated care in chronic disease management? Its main roles have been in providing education (to improve self-management), in enabling information transfer (e.g. telemonitoring), in facilitating contact with health professionals (e.g. telephone support and follow-up) and in improving electronic records. That is, telemedicine has been used in both the process of care and the outcome of care.

Note that the term “telemedicine” has a wide definition – medicine practised at a distance – and a correspondingly wide range of telemedicine applications has been trialled in the management of chronic diseases. The telemedicine interactions have been of two types, either taking place in real time (e.g. videoconferencing) or asynchronously (e.g. store-and-forward transmission of data from a home glucose meter). Monitoring applications have been entirely automatic (e.g. passive monitoring of activity using room sensors) or have required the patient to do something (e.g. transmit bodyweight values using the buttons on a telephone). Educational applications have employed specially designed home devices, or depended on web access from PCs or smart phones.

**Figure 1:**
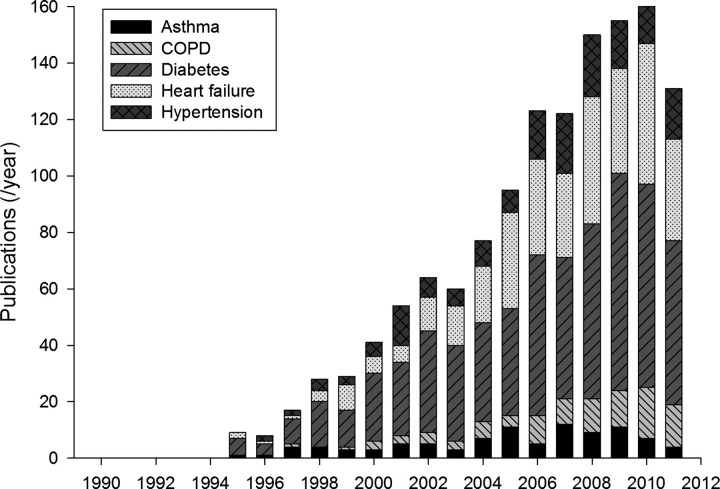
Medline publications on telemedicine and five chronic diseases. There were 1324 publications between 1990 and 2011.

A review conducted in 2003 concluded that telemedicine looked promising for chronic disease management, but that good quality studies were scarce and that the generalizability of most findings was rather limited.^5^ What has changed in the ensuing nine years? First, experimentation with telemedicine has continued apace. There has been a continued increase in the publication of papers concerning telemedicine and chronic diseases (Figure 1). The numbers of papers has increased approximately five-fold since 2003. Second, there have been some substantial implementations. For example, the Veterans Administration in the US has reported some 50,000 patients managed with home telecare.^6^ Despite this enthusiasm, almost nothing is known about the cost-effectiveness of telemedicine in chronic disease management.

Cost-effectiveness is a critical matter for the adoption of any new technique or technology into health care. The conventional approach to answering questions about cost-effectiveness is to summarize the results of randomized controlled trials (RCTs) and produce a pooled estimate of effect, by conducting a meta-analysis. Generally, the effect of interest is the Quality-Adjusted Life Year (QALY). If such an estimate for the cost of a QALY passes an agreed threshold (e.g. £25-35,000 in the UK NHS), then widespread implementation of the intervention is likely. Ultimately, if telemedicine is going to be used on a wide scale in public healthcare systems, it will need to pass tests such as these. However, there are significant difficulties in taking this approach in the present context. Crucially, there have been very few studies of cost-effectiveness, so calculating a pooled estimate is impossible.

Since estimating the cost-effectiveness is unfeasible because of the lack of data, some lesser assessment of the value of telemedicine may be the best that can be managed for the time being. Again, the conventional approach is a meta-analysis, examining a quantitative outcome such as mortality, emergency department visits or length of stay in hospital. Such analyses have indeed been conducted for specific outcomes in certain chronic diseases. Here the problem is that the published trials have employed a wide range of outcome measures, so that a pooled estimate of any one outcome reduces the size of the dataset very considerably. For example, there are at least 11 RCTs of telemedicine in COPD, but the published estimate of the risk ratio for mortality was based on only three studies.^7^

Since conventional meta-analysis cannot yet provide a robust summary of this very heterogeneous field, a different procedure must be used if the value of telemedicine in chronic disease management is to be estimated. The present study therefore takes a new approach, in order to obtain a high-level view of the value of telemedicine in chronic disease management.

## Methods

The analysis was confined to RCTs in which one or more telemedicine interventions had been compared with a control group. It was restricted to patients with one of the following common chronic diseases: asthma, COPD, diabetes, heart failure, hypertension. The telemedicine intervention could include telephone support, telemonitoring, videoconferencing, etc. The “value” of the trial result was defined in terms of the outcomes specified by the investigators in each study individually. A synthesis was carried out by meta-regression.

### Identification of studies

The studies of interest were RCTs concerning the use of telemedicine in chronic disease. Candidate studies were identified in a three stage process. First, systematic reviews of the use of telemedicine in the chronic diseases of interest were identified, and the reference lists of these reviews were searched by hand. Second, a computerised literature search was conducted to identify individual RCTs directly. Finally, the reference lists of included studies were searched by hand.

### Computerized searching

Computerized searches of the Medline database were conducted in July/August 2011 to identify systematic reviews, and to identify RCTs. The search terms were:

1. Telemedicine AND;
2. Randomized controlled trial/systematic review AND;
3. Asthma OR COPD OR diabetes OR heart failure OR hypertension.

Non-English language papers were included.

### Selection of studies

Candidate studies were selected for further examination based on the abstract; full copies of the articles were then examined to confirm that they met the following inclusion criteria. Studies were included if they reported:

1. An RCT;
2. A telemedicine intervention, such as telemonitoring or telephone support;
3. Patients with a single chronic disease, or if multiple disease groups had been studied, then the results had to be separately reported for each disease group of interest.

### Data extraction

The following information was extracted from each of the studies:

No of subjects
Type of patient, e.g. disease and severity
Nature of the intervention. In addition, details were recorded about whether there was-
  Routine voice contact with a person such as a case manager, nurse specialist or pharmacist
  Voice contact with an interactive voice response (IVR) system
  Video contact with a health professional, e.g. videoconferencing
  Messaging with a health professional, e.g. using email, web messaging or online chat
  Telemonitoring, e.g. automatic transmission of data such as symptoms or vital signs
Duration (months)
Primary and other outcomes
Result
Overall value of intervention.

The overall value of the intervention was rated in terms of the outcomes specified for the study in question, with the effect categorised on a 5-point scale, see Table 1.

**Table 1:** Categorisation of the value of the intervention

Value	Criterion	Score
Positive	Primary outcome significantly better (*P* < 0.05) in the intervention group compared to control	5
Weakly positive	One or more secondary outcomes significantly better, if the primary outcome was not significantly better	4
No effect	No significant difference between intervention and control groups	3
Weakly negative	One or more secondary outcomes significantly worse, if the primary outcome was not significantly worse	2
Negative	Primary outcome significantly worse in the intervention group compared to control	1

### Synthesis

The relation between the value of the interventions trialled (i.e. the estimates of effect) and various potential explanatory variables was first examined graphically. Possible predictors were then examined collectively using regression modelling. Since the dependent variable was categorical, an ordered logit regression was employed, using a standard package (Gretl. See http://gretl.sourceforge.net/).

## Results

### Identification and selection of studies

In the first stage of the identification process, a total of 22 systematic reviews was identified relating to the use of telemedicine in the chronic diseases of interest.^7–28^ In the second stage, a total of 264 reports of RCTs was identified. After screening these papers and examining the reference lists of those included, there was a final total of 141 RCTs which met the inclusion criteria, see Table 2. These papers reported trials of 148 interventions, i.e. some trials had multiple experimental arms.

**Table 2:** Identification and selection of studies

	Asthma	COPD	Diabetes	Heart failure	Hypertension	Total
Systematic reviews	1	5*	6	9	1	22
No of papers retrieved in initial search	21	20	106	75	42	264
No of papers included in the present study	20	11	39	57	14	141
No of interventions**	20	11	39	61	17	148

*one review concentrated on the organizational process, rather than health care outcomes^9^

**some trials had multiple experimental arms

### RCTs and interventions trialled

In asthma, trials of 20 interventions were identified, see Table 3 (see online only supplementary data: http://jtt.rsmjournals.com/lookup/suppl/doi:10.1258/jtt.2012.120219/-/DC1). These trials involved a total of 10,406 patients. Outcome measures commonly employed were healthcare utilization, symptoms and quality of life.

In COPD, trials of 11 interventions were identified; two articles contained the details about one trial, see Table 4 (see online only supplementary data: http://jtt.rsmjournals.com/lookup/suppl/doi:10.1258/jtt.2012.120219/-/DC1). The trials involved a total of 1104 patients. Outcome measures commonly employed were hospital admissions and quality of life.

In diabetes, trials of 39 interventions were identified; there were three trials in which the details were contained in two reports each, see Table 5 (see online only supplementary data: http://jtt.rsmjournals.com/lookup/suppl/doi:10.1258/jtt.2012.120219/-/DC1). The trials involved a total of 4970 patients. Outcome measures commonly employed were HbA_1c_, quality of life and self-efficacy.

In heart failure, trials of 61 interventions were identified; there were six trials in which the details were contained in two reports each, see Table 6 (see online only supplementary data: http://jtt.rsmjournals.com/lookup/suppl/doi:10.1258/jtt.2012.120219/-/DC1). The trials involved a total of 16,388 patients. Outcome measures commonly employed were mortality, hospital admissions, quality of life and healthcare costs.

In hypertension, trials of 17 interventions were identified – there were 14 RCTs, see Table 7 (see online only supplementary data: http://jtt.rsmjournals.com/lookup/suppl/doi:10.1258/jtt.2012.120219/-/DC1). These trials involved a total of 4827 patients. Outcome measures commonly employed were blood pressure and healthcare costs.

### Size of the trials

**Figure 2:**
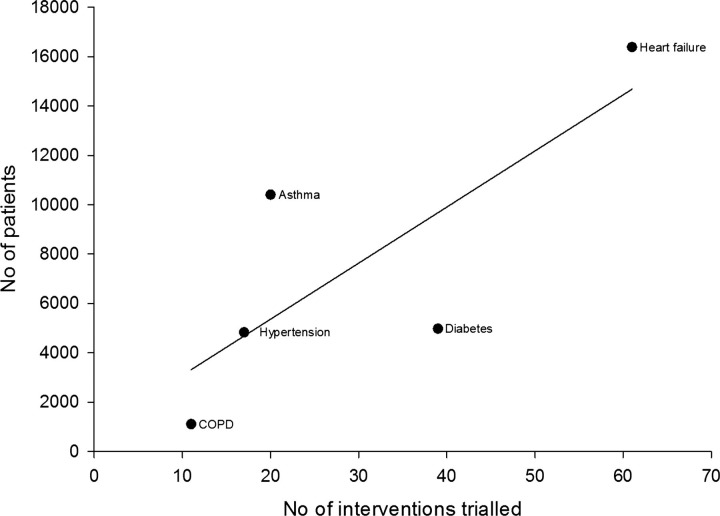
Size of the trials. The solid line shows the linear regression

The 141 RCTs involved a total of 37,695 patients, i.e. an average trial size of about 270 patients. There was a tendency for the trials of patients with diabetes to be slightly smaller than the norm, and for the trials of patients with asthma to be slightly larger than the norm, see Figure 2. There was a linear relationship, though not significant, between the numbers of interventions trialled and the total number of patients in those trials.

### Effect estimates

Most studies reported favourable effects: either positive (*n* = 65) or weakly positive (*n* = 43) in terms of the outcomes specified by the investigators in their trials. There were 38 studies in which the intervention was not significantly different from the control, and only two (one weakly negative and one negative) in which the intervention was worse than the control. That is, 73% of studies were favourable to the intervention, 26% were neutral, and 1% were unfavourable.

### Heterogeneity

**Figure 3:**
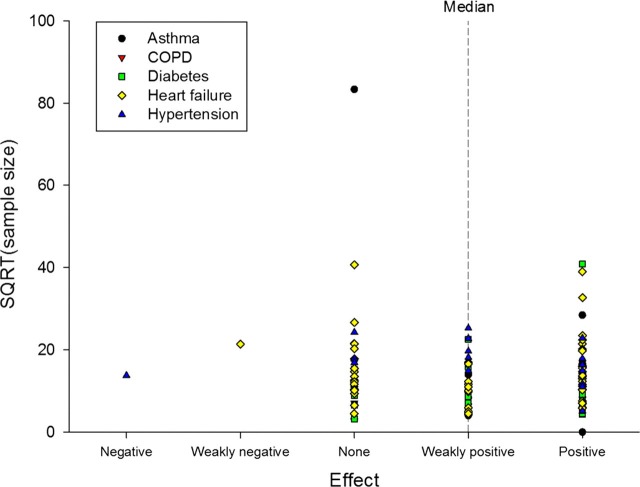
Funnel plot

In the absence of bias and between-study heterogeneity, the scatter in the effect estimates will be due to sampling variation alone. A plot of the effect estimates from individual studies against some measure of the precision of each study will resemble a symmetrical inverted funnel. Using the square root of the sample size as an estimate of precision showed that there was considerable heterogeneity in the dataset, see Figure 3.

### Potential explanatory variables

**Figure 4:**
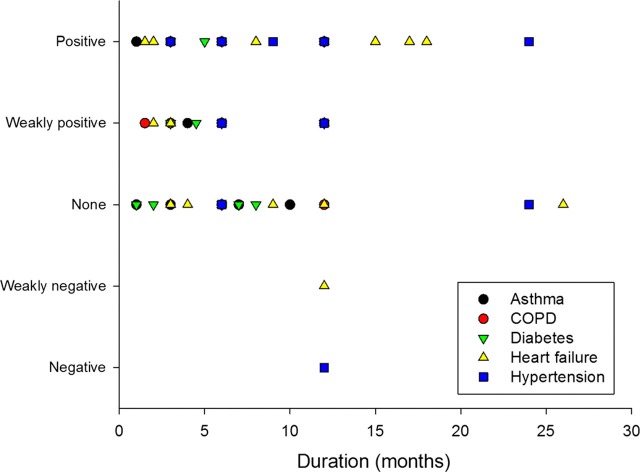
Duration of interventions

The relation between the duration of the intervention and the effect is shown in Figure 4. There was no obvious tendency for interventions applied for longer periods to produce more positive results. An ordered logit regression was not significant.

**Figure 5:**
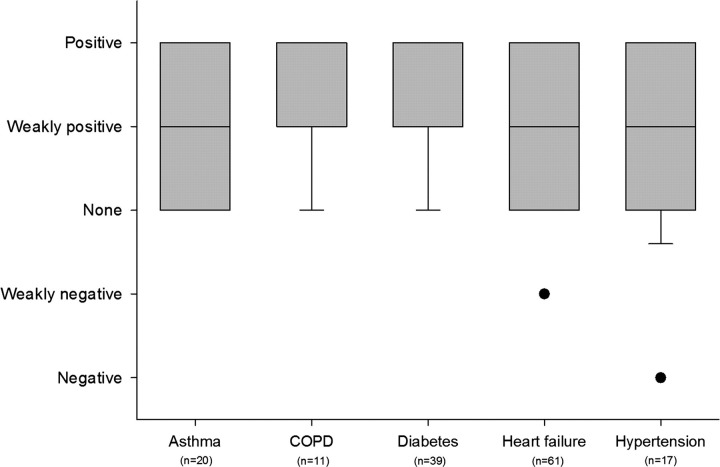
Disease type. The boundaries of the boxes indicate the 25th and 75th percentiles, and a line within the box marks the median. The whiskers (error bars) above and below the boxes indicate the 90th and 10th percentiles. Potential outliers are shown individually

The median effect in all five chronic diseases was weakly positive. In the individual diseases, the median effect was weakly positive for asthma, diabetes, heart failure and hypertension, and positive for COPD. The effect in different disease types is summarised in Figure 5. There were no significant differences between the different disease types (Kruskal Wallis *P* = 0.96).

**Figure 6:**
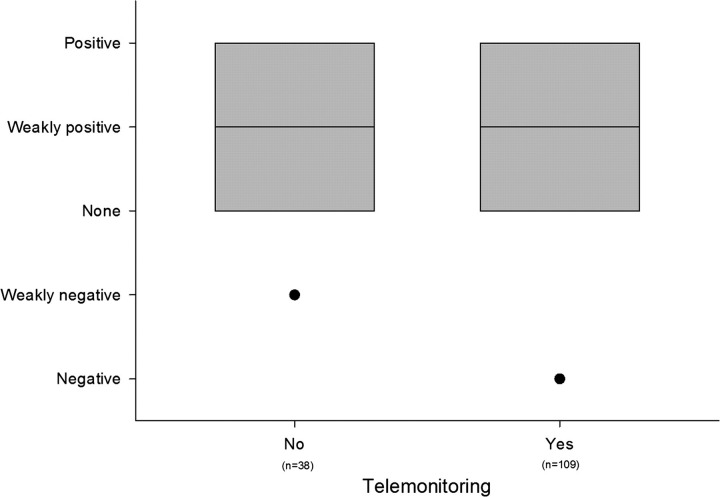
Telemonitoring. Box plot attributes as for Figure 5

**Figure 7:**
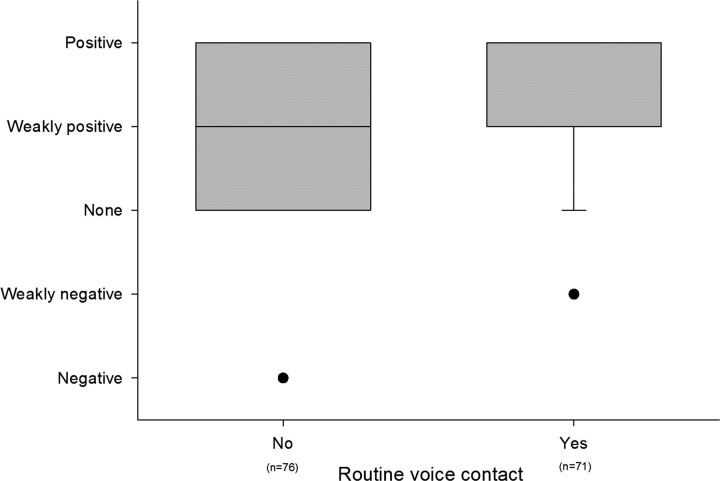
Routine voice contact. Box plot attributes as for Figure 5

**Figure 8:**
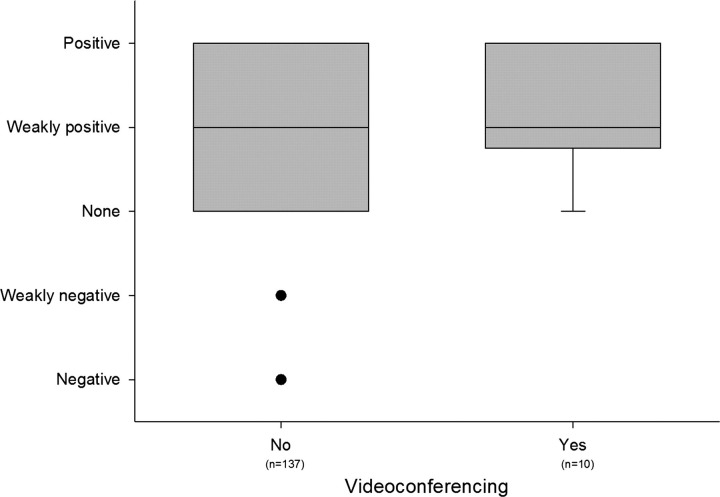
Videoconferencing. Box plot attributes as for Figure 5

The effect in trials using telemonitoring is shown in Figure 6; there was no significant difference in effect between interventions which employed telemonitoring and those which did not. The effect in trials using routine voice contact is shown in Figure 7; there was no significant difference in effect between interventions which employed routine voice contact and those which did not. The effect in trials using videoconferencing is shown in Figure 8; there was no significant difference in effect between interventions which employed videoconferencing and those which did not.

**Figure 9:**
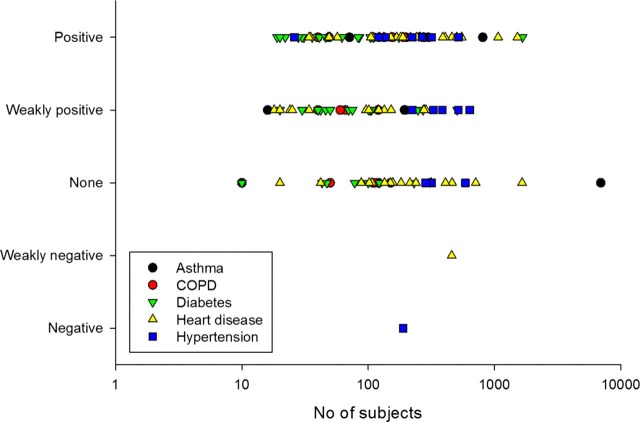
Number of subjects

The relation between the number of subjects and the effect is shown in Figure 9. There was no obvious tendency for trials in which large numbers of subjects had been employed to produce more positive results. An ordered logit regression was not significant.

**Figure 10:**
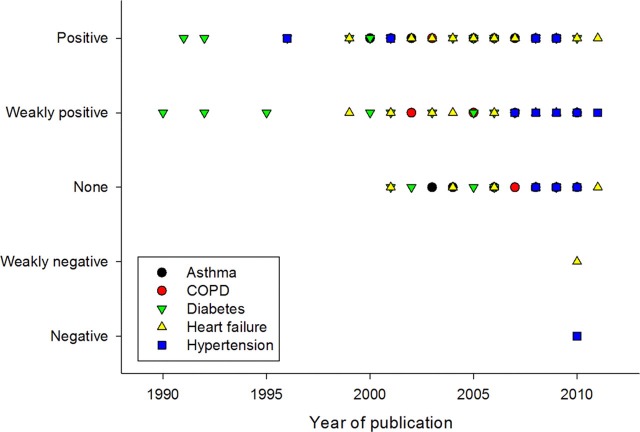
Year of publication

The relation between the year of publication and the effect is shown in Figure 10. There was a tendency for studies which had been published earlier to report more positive findings. An ordered logit regression was significant at *P* < 0.05.

### Synthesis

In an ordered logit regression with all possible predictors, none was significant except year of publication (*P* = 0.02).

## Discussion

During the last 20 years, more than 1300 Medline papers have been published concerning the use of telemedicine in the five chronic diseases considered here. Approximately one in ten of these studies have been formal randomised trials. A wide range of outcomes has been reported in these experiments, which makes a conventional meta-analysis of the entire dataset unfeasible. The present work was undertaken in order to obtain a high-level overview of the value of telemedicine in chronic disease management in a general sense. In doing so, data were considered from RCTs in which more than one intervention had been trialled.

### Multiplicity adjustment

In a trial with multiple experimental arms, the question arises whether a multiplicity adjustment is required to reduce the probability of a false-positive result, i.e. with two treatment arms, each intervention group will be compared separately with the same control group. One possibility, for example, would be to use a Bonferroni adjustment. However, the consensus of opinion is that a multiplicity adjustment would not be necessary if the aim of the trial was to answer questions about the efficacy of each intervention separately, i.e. the interpretation of the results of one comparison had no direct bearing on the interpretation of the results of the others.^29^ In the present context, the multi-arm studies found in the review investigated interventions such as telephone support and telemonitoring, which can be considered independent. Thus a multiplicity adjustment was not required for the present analysis.

### Systematic reviews

During the identification of RCTs for the present study, a total of 22 systematic reviews was identified concerning the use of telemedicine in the five chronic diseases of interest. In approximately half of these reviews, the authors provided a qualitative summary of the value of telemedicine, usually in the form of a narrative review; none of these concluded negatively, i.e. that telemedicine was unhelpful in chronic disease management, see Table 8.

**Table 8:** Systematic reviews reporting pooled estimates of quantitative outcomes (“NS difference” indicates no difference between intervention and control groups at *P* ≥ 0.05; “significant improvement” indicates that there were significantly better outcomes in the intervention group at *P* < 0.05)

	Comment	Quality of life	Emergency department visits	Hospitalizations*	Mortality*	HbA_1c_	Hypoglycaemia	Ketoacidosis	Qualitative conclusion
**Asthma**									
McLean 2010^21^		Significant improvement (but not a clinically important difference)	NS difference	Significant improvement					

**COPD**									
Bartoli 2009^9^	(review of the organizational process, rather than health care outcomes)								
Bolton 2010^10^									Neutral
Jaana 2009^18^									Neutral
McLean 2011^7^		Significant improvement	Significant improvement	Significant improvement	NS difference				
Polisena 2010^23^					NS difference				

**Diabetes**									
Costa 2009^14^									Neutral
Farmer 2005^16^						NS difference			
Polisena 2009^22^						Significant improvement			
Shulman 2010^26^						NS difference	NS difference	NS difference	
Verhoeven 2007^27^						NS difference			
Verhoeven 2010^28^						NS difference			

**Heart failure**									
Chaudhry 2007^11^									Neutral
Clark 2007^12^				Significant improvement	Significant improvement				
Clarke 2011^13^			NS difference	Significant improvement	Significant improvement				
Dang 2009^15^									Weakly positive
Inglis 2010^17^				Significant improvement	Significant improvement				
Louis 2003^19^									Weakly positive
Martínez 2006^20^									Weakly positive
Polisena 2010^24^					Significant improvement				
Seto 2008^25^	Cost analysis								Positive

**Hypertension**									
AbuDagga 2010^8^									Positive

*sometimes subdivided into all-cause and that due to the disease in question

The other half of the reviews provided pooled estimates of various quantitative outcomes. There were four quantitative outcomes which were potentially applicable in all five diseases:

1. Quality of life;
2. Emergency department visits;
3. Hospitalization;
4. Mortality.

In addition, there were three quantitative outcomes which were specific to diabetes:

1. HbA_1c_;
2. Severe hypoglycaemia;
3. Diabetic ketoacidosis.

Between them, the 12 systematic reviews provided 23 pooled estimates of effect, of which approximately half showed telemedicine to provide significantly better outcomes than the control condition. Conversely, the other half of the pooled estimates showed telemedicine to be no better than the control condition. This emphasises the rather weak and unsatisfactory conclusions which can be drawn from the systematic reviews presently available.

### Heart failure

Of the 22 systematic reviews identified, the largest number (9) concerned the use of telemedicine in heart failure. These reviews, which were published over a nine-year period, provide eight pooled estimates of effect, all except one being significantly positive in favour of telemedicine. Of all the chronic diseases considered in the present study, therefore, the evidence would appear most favourable for heart failure. Indeed, the appearance of an authoritative Cochrane review that favoured the use of telemedicine (telephone support or telemonitoring) in heart failure^17^ would normally signal acceptance of efficacy by the scientific community and potentially pave the way for widespread trials of effectiveness. Unfortunately, there have been two subsequent reports^30,31^ from large, well-powered RCTs which are contradictory, and at the time of writing, we expect the Cochrane review to be revised and re-issued to reflect this.

### Overview

To avoid the problem of requiring a common outcome from all trials, as would be needed for a conventional meta-analysis, the present study adopted a different approach in which the value of each intervention was categorised in terms of the outcomes specified by the investigators in that trial. From this, it can be seen that the majority of trials report positive effects, i.e. there is a strong suspicion of publication bias. This is supported by the observation that more recent publications tend to report weaker effects. Publication bias was also suggested by the asymmetric funnel plot for the dataset. While this is certainly a plausible explanation of the overwhelmingly positive findings reported, it is not the only one.^32^ There is likely to be true heterogeneity because of differences between the interventions and differences between the diseases.

The present review suggests that there are no major differences in the value of the telemedicine intervention between the disease types. Furthermore, neither telemonitoring nor videoconferencing appear to be superior to telephone support. Most studies have been relatively short-term which, in the case of chronic diseases, may weaken their power to demonstrate an effect. The work of Shea *et al.*,^33^ who reported 5-year follow-up in patients with diabetes, demonstrates that long-term telemedicine interventions are possible.

A wide range of outcomes has been employed in the trials reviewed. However, there have been few studies in which cost-effectiveness has been measured. The work by Hebert *et al.* ^34^ reporting QALY data for telemedicine in heart failure, therefore represents an exemplar.

On the basis of the work reviewed, it is not possible to state that telemedicine of a particular type will be cost-effective in the management of one or more chronic diseases. After nearly 20 years of randomised trials work, this seems both surprising and disappointing. Nonetheless, the majority of the studies conducted have reported positive effects in terms of the outcomes specified in those trials. This raises the possibility that the beneficial effect reported is not due to telemedicine itself, so much as to the increased attention due to the experimental intervention, i.e. that a Hawthorne effect is at least partly responsible. Future work should be designed to separate the true effects of telemedicine from putative placebo effects.

### Limitations

The present study had certain limitations. First, although the search for studies was conducted largely in accordance with the procedure for a systematic review, there may be other RCTs that could have been found. Second, the value of each study was assumed to be the same in the meta-regression, i.e. no attempt was made to weight the studies. Third, other chronic diseases may be of interest in addition to the five common chronic diseases which were studied. Finally, synthesizing disparate outcomes data in different diseases has not been attempted before in telemedicine work, so far as I am aware. It is an acknowledged limitation of this new approach that the theoretical foundation remains to be developed. In the meantime, it can at least be regarded as a qualitative technique.

### Future work

The present study raises a number of questions about the intrinsic value of telemedicine in the management of chronic disease. It would therefore be useful if future studies were designed very carefully, in order to identify the true value of distance support. It would also be valuable to future reviewers if a minimum dataset could be agreed for the outcome measures. Quantitative indices, from which pooled estimates of effect can be calculated, and which are applicable across all disease groups include:

1. Quality of life (as measured on the scale appropriate to the disease in question);
2. Cost to society;
3. Emergency department visits;
4. Days in hospital;
5. Mortality.

The last three may be further categorised as “all-cause” or disease-specific.

Finally, it seems unlikely that in a chronic disease, any intervention can have much effect unless applied for a long period. Future studies might consider interventions lasting years rather than months.

### Conclusion

The evidence base for the value of telemedicine in managing chronic diseases is on the whole weak and contradictory.

## Supplementary Material

Supplementary material
