# Supplementary material for: Twenty years of telemedicine in chronic disease management – an evidence synthesis
Source: J Telemed Telecare. 2012 Jun;18(4):211–20. doi: 10.1258/jtt.2012.120219 (PMC3366107; doi:10.1258/jtt.2012.120219)
Supplement: Supplementary material [file JTT-12-02-019_Supplementary.pdf]

APPENDIX

Table 3 Asthma RCTs

Study	No of subjects	Type of pt	Intervention	Telemonitoring?	Duration (months)	Outcomes	Result	Overall value of intervention
Bynum 2001	49	Asthma	Inhaler tuition ("telepharmacy counselling") given by videoconf on 3 occasions at schools; control received written instructions	N	1	Inhaler technique checklist	Sig improvement on technique in T/M group compared to control	POSITIVE
Chan 2007	120	Persistent asthma	Web-based interaction, including uploading of home videos of inhaler and PF technique	Y	12	Adherence; Inhaler technique; QoL; Asthma knowledge	Significantly better inhaler technique than controls; better submission of asthma diary data	MILDLY POSITIVE
Chan 2003	10	Persistent asthma	Web-based education; web diary and uploading of data/videos	Y	6	Adherence; Symptoms; QoL; Asthma knowledge	No differences	NONE
Chatkin 2006	271	Persistent asthma	Telephone calls every 2 weeks from nurse educator	N	3	Adherence	Sig higher adherence in T/M group	POSITIVE
Clark 2007	808	Women with asthma	Telephone counselling (6 calls) from nurse educator	N	12	Nights with symptoms; Days off work/school; ED visits; GP visits	Sig better results in all outcomes for T/M group	POSITIVE
de Jongste 2009	151	Children with asthma	Airway inflammation monitor and symptom diary – results sent daily to monitoring centre	Y	7	Symptom-free days; Symptom scores; Medication dose; Lung function	No sig diff in symptom-free days between T/M and control group No diff in other outcomes	NONE
Donald 2008	71	Asthma	Weekly telephone calls from asthma educators	N	12	Hospital admissions; Various secondary outcomes	Sig fewer admissions in T/M group than control group; most secondary outcomes NS	POSITIVE
Gruffydd-Jones 2005	194	Asthma	Telephone review by asthma nurse every 6 months	N	12	ACQ, Asthma Control Questionnaire; QoL; Costs	No sig change in ACQ in T/M group compared to control; Sig improvement in QoL; Sig reduction in costs	MILDLY POSITIVE
Guendelman 2002	134	Asthma	Daily use of electronic diary (Health Buddy) for symptoms, peak flow readings etc with automatic transmission to nurse	Y	3	Limitations in activity; Symptoms; Absence from school; Yellow/Red zone PEF readings	Significant improvement in limitations-of-activity, and low PEF readings compared to control group	POSITIVE
Jan 2007	196	Persistent asthma	Electronic diary with upload of spirometer data via PC	Y	3	QoL; Asthma knowledge	Significantly improved symptoms and QoL in T/M group compared to control group	MILDLY POSITIVE
Khan 2004	310	Discharged from ED following asthma	Follow-up telephone consultation with asthma educator	N	1	Number of days of wheezing	No sig diff between T/M and control group	NONE
Kokubu 1999 & Kokobu 2000	Unclear	High-risk asthma	Telephone advice from nurse	Unclear	6	Hospitalization	Sig reduction in hospitalizations in T/M group compared with control	POSITIVE
Ostojic 2005	16	Moderate persistent asthma	Daily SMS transmission of PF data	Y	4	Symptoms; Lung function	Significantly better symptom scores compared to controls; sig better FEV1	MILDLY POSITIVE
Pinnock 2005	278	Symptomatic asthma	Telephone review. Control patients were offered FTF review	N	3	Whether patient reviewed within 3 months; Cost	Sig more T/M patients reviewed than control; NS difference in cost per consultation	POSITIVE
Prabhakaran 2010	120	Asthma	SMS monitoring and education	Y	3	ACT, Asthma Control Test; No of nebulizations; ED visits	No sig differences	NONE
Rasmussen 2005	300	Definite asthma	Electronic diary with upload of spirometer data either via telephone or via web Decision-support tool for doctors	Y	6	Symptoms; Asthma QoL; FEV1; Airway hyper-responsiveness	Significantly improved symptoms, QoL and FEV1 compared to groups treated by specialists or by GPs	POSITIVE

(Continued)

Table 3 Continued

Study	No of subjects	Type of pt	Intervention	Telemonitoring?	Duration (months)	Outcomes	Result	Overall value of intervention
van der Meer 2009	200	Doctor-diagnosed asthma	Daily FEV and symptoms reported via the web	Y	12	Asthma QoL; Asthma control	QoL sig better in T/M group compared with controls; Some control outcomes better	POSITIVE
Vollmer 2006	6948	Asthma	Three automated telephone calls (IVR system) approx 5 months apart	N	10	Healthcare utilization; Medication use; QoL	NS diffs	NONE
Willems 2008	109	Asthma	Electronic spirometer uploaded data every month	Y	12	QoL; Symptoms; Medication	NS differences	NONE
Xu 2010	121	Doctor-diagnosed asthma	One group had telephone/email support from asthma nurse. One group had IVR support	Y	6	Health resource utilization; QoL	NS diffs in QoL between groups; Lower costs in the two T/M groups (but not sig)	NONE

Asthma RCTs

Bynum A, Hopkins D, Thomas A, Copeland N, Irwin C. The effect of telepharmacy counseling on metered-dose inhaler technique among adolescents with asthma in rural Arkansas. *Telemed J E Health* 2001;**7**:207–217
Chan DS, Callahan CW, Hatch-Pigott VB, *et al.* Internet-based home monitoring and education of children with asthma is comparable to ideal office-based care: results of a 1-year asthma in-home monitoring trial. *Pediatrics* 2007;**119**:569–578
Chan DS, Callahan CW, Sheets SJ, Moreno CN, Malone FJ. An Internet-based store-and-forward video home telehealth system for improving asthma outcomes in children. *Am J Health Syst Pharm* 2003;**60**:1976–1981
Chatkin JM, Blanco DC, Scaglia N, Wagner MB, Fritscher CC. Impact of a low-cost and simple intervention in enhancing treatment adherence in a Brazilian asthma sample. *J Asthma* 2006;**43**:263–266
Clark NM, Gong ZM, Wang SJ, Lin X, Bria WF, Johnson TR. A randomized trial of a self-regulation intervention for women with asthma. *Chest* 2007;**132**:88–97
Donald KJ, McBurney H, Teichtahl H, Irving L. A pilot study of telephone based asthma management. *Aust Fam Physician* 2008;**37**:170–173
Gruffydd-Jones K, Hollinghurst S, Ward S, Taylor G. Targeted routine asthma care in general practice using telephone triage. *Br J Gen Pract* 2005;**55**:918–923
Guendelman S, Meade K, Benson M, Chen YQ, Samuels S. Improving asthma outcomes and self-management behaviors of inner-city children: a randomized trial of the Health Buddy interactive device and an asthma diary. *Arch Pediatr Adolesc Med* 2002;**156**:114–120
Jan R-L, Wang J-Y, Huang M-C, Tseng S-M, Su H-J, Liu L-F. An internet-based interactive telemonitoring system for improving childhood asthma outcomes in Taiwan. *Telemed J E Health* 2007;**13**:257–268
de Jongste JC, Carraro S, Hop WC, *et al.* Daily telemonitoring of exhaled nitric oxide and symptoms in the treatment of childhood asthma. *Am J Respir Crit Care Med* 2009;**179**:93–97
Khan MSR, O'Meara M, Stevermuer TL, Henry RL. Randomized controlled trial of asthma education after discharge from an emergency department. *J Paediatr Child Health* 2004;**40**:674–677
Kokubu F, Nakajima S, Ito K, *et al.* [Hospitalization reduction by an asthma tele-medicine system]. *Arerugi* 2000;**49**:19–31 [Japanese]
van der Meer V, Bakker MJ, van den Hout WB, *et al.* Internet-based self-management plus education compared with usual care in asthma: a randomized trial. *Ann Intern Med* 2009;**151**:110–120
Ostojic V, Cvoriscec B, Ostojic SB, Reznikoff D, Stipic-Markovic A, Tudjman Z. Improving asthma control through telemedicine: a study of short-message service. *Telemed J E Health* 2005;**11**:28–35
Pinnock H, McKenzie L, Price D, Sheikh A. Cost-effectiveness of telephone or surgery asthma reviews: economic analysis of a randomised controlled trial. *Br J Gen Pract* 2005;**55**:119–124
Prabhakaran L, Chee WY, Chua KC, Abisheganaden J, Wong WM. The use of text messaging to improve asthma control: a pilot study using the mobile phone short messaging service (SMS). *J Telemed Telecare* 2010;**16**:286–290
Rasmussen LM, Phanareth K, Nolte H, Backer V. Internet-based monitoring of asthma: a long-term, randomized clinical study of 300 asthmatic subjects. *J Allergy Clin Immunol* 2005;**115**:1137–1142
Vollmer WM, Kirshner M, Peters D, *et al.* Use and impact of an automated telephone outreach system for asthma in a managed care setting. *Am J Manag Care* 2006;**12**:725–733
Willems DCM, Joore MA, Hendriks JJE, Nieman FHM, Severens JL, Wouters EFM. The effectiveness of nurse-led telemonitoring of asthma: results of a randomized controlled trial. *J Eval Clin Pract* 2008;**14**:600–609
Xu C, Jackson M, Scuffham PA, *et al.* A randomized controlled trial of an interactive voice response telephone system and specialist nurse support for childhood asthma management. *J Asthma* 2010;**47**:768–773

Table 4 COPD RCTs

Study	No of subjects	Type of patient	Intervention	Telemonitoring?	Duration (months)	Outcomes	Result	Overall value of intervention
Bourbeau 2003	191	Hospitalized acutely at least once in previous year	Educational programme; monthly telephone calls to follow-up	N	12	Hospital admissions; Doctor visits; QoL; Lung function;	Sig fewer hospitalizations in T/M group than controls; fewer ED visits; fewer unscheduled GP visits; improved QoL; no change in lung function	POSITIVE
Casas 2006	155	Previous hospitalization for at least 2 days (24% were on LTOT)	Educational programme; web-based call centre; Patient-initiated and scheduled telephone calls	N	12 (max)	Hospital admissions; Survival	Lower hospitalization rate; fewer admissions. No change in mortality	POSITIVE
de Toledo 2006	157	COPD	Nurse home visit with telemetry; web-based education	Sort of	12	Hospitalizations; ED visits; Mortality	Reduced hospitalizations	POSITIVE n.b. the patients didn't do telemonitoring themselves (it was the nurses during home visits)
Egan 2002	66	FEV < 50%	Case manager; regular telephone calls	N	1.5	QoL; Hospitalizations	Improved QoL. No change in re-admissions	MILDLY POSITIVE
Farrero 2001	122	LTOT	Monthly telephone call plus quarterly home nurse visits	N	12	ED visits; Hospitalizations; Costs; QoL	Sig fewer ED visits, hospitalizations in T/M group compared with controls; Lower costs; no diff in QoL	POSITIVE
Garcia-Aymerich 2007	113	COPD	Individualized care post-discharge. Six teletelephone calls from nurse specialist. Web-based call centre	N	12	Clinical status; QoL	NS differences in clinical status or QoL	NONE
Koff 2009	40	COPD stage 3 or 4	Educational programme; home monitoring (Health Buddy)	Y	3	QoL; Healthcare costs; Exacerbations	Sig improvement in QoL in T/M group compared to controls; costs were lower but NS	POSITIVE
Lewis 2010a, b	40	mod/severe COPD	Daily telemonitoring (Q&A, temp, oximetry)	Y	6	QoL; Hospital admissions; ER visits; Primary care contacts	NS diff in QoL between T/M and control; No diff in admissions or ER visits; Sig fewer primary care contacts	MILDLY POSITIVE
Nguyen 2008	50	COPD with mild impaired FEV1	Symptom and exercise data sent via PDA to nurse at time of exercise	Y	6	Dyspnoea; Exercise; QoL	NS diff between groups in primary outcome; Marginal improvement in exercise performance; NS diffs in other outcomes	NONE
Vitacca 2009	240 (110 with COPD)	COPD (LTOT)	Scheduled telephone calls with nurse; PSTN transmission of SpO2 data on request	On request	12	Hospitalizations; Exacerbations; GP calls; Costs	Sig fewer hospitalizations, exacerbations, GP visits in T/M group than controls; lower costs (but NS?)	POSITIVE
Wong 2005	60	COPD	Two follow-up telephone calls	N	3	Self-efficacy; Health service use	Self-efficacy improved; No change in health service use	MILDLY POSITIVE

COPD RCTs

Bourbeau J, Julien M, Maltais F, *et al.* Reduction of hospital utilization in patients with chronic obstructive pulmonary disease: a disease-specific self-management intervention. *Arch Intern Med* 2003; **163**: 585–591

Casas A, Troosters T, Garcia-Aymerich J, *et al.* Integrated care prevents hospitalisations for exacerbations in COPD patients. *Eur Respir J* 2006;**28**:123–130

Egan E, Clavarino A, Burridge L, Teuwen M, White E. A randomized control trial of nursing-based case management for patients with chronic obstructive pulmonary disease. *Lippincotts Case Manag* 2002;**7**:170–179

Farrero E, Escarrabill J, Prats E, Maderal M, Manresa F. Impact of a hospital-based home-care program on the management of COPD patients receiving long-term oxygen therapy. *Chest* 2001;**119**:364–369

Garcia-Aymerich J, Hernandez C, Alonso A, *et al.* Effects of an integrated care intervention on risk factors of COPD readmission. *Respir Med* 2007;**101**:1462–1469

Koff PB, Jones RH, Cashman JM, Voelkel NF, Vandivier RW. Proactive integrated care improves quality of life in patients with COPD. *Eur Respir J* 2009;**33**:1031–1038

Lewis KE, Annandale JA, Warm DL, Hurlin C, Lewis MJ, Lewis L. Home telemonitoring and quality of life in stable, optimised chronic obstructive pulmonary disease. *J Telemed Telecare* 2010;**16**:253–259

Lewis KE, Annandale JA, Warm DL, *et al.* Does home telemonitoring after pulmonary rehabilitation reduce healthcare use in optimized COPD? A pilot randomized trial. *COPD* 2010;**7**:44–50

Nguyen HQ, Donesky-Cuenco D, Wolpin S, *et al.* Randomized controlled trial of an internet-based versus face-to-face dyspnea self-management program for patients with chronic obstructive pulmonary disease: pilot study. *J Med Internet Res* 2008;**10**:e9

de Toledo P, Jiménez S, del Pozo F, Roca J, Alonso A, Hernandez C. Telemedicine experience for chronic care in COPD. *IEEE Trans Inf Technol Biomed* 2006;**10**:567–573

Vitacca M, Bianchi L, Guerra A, *et al.* Tele-assistance in chronic respiratory failure patients: a randomised clinical trial. *Eur Respir J* 2009;**33**:411–418

Wong KW, Wong FKY, Chan MF. Effects of nurse-initiated telephone follow-up on self-efficacy among patients with chronic obstructive pulmonary disease. *J Adv Nurs* 2005;**49**:210–222

Table 5 Diabetes RCTs

Study	No of subjects	Type of pt	Intervention	Telemonitoring?	Duration (months)	Outcomes	Result	Overall value of intervention (on HbA1c)
Ahring 1992	42	HbA1c ≥7%	BG measured 5 times/d and uploaded via modem each week	Y	3	HbA1c	NS difference between T/M group and control at end of study, but sig difference after 6 weeks	MILDLY POSITIVE
Bellazzi 2003	104	Type 1 and Type 2	BG data transmitted by modem; Web access and IVR system	Y	6	HbA1c; Variance in HbA1c	NS diff in HbA1c in T/M group compared to control; Sig less variance in HbA1c	MILDLY POSITIVE
Bergenstal 2005	47	Type 1 and Type 2	BG data transmitted approx weekly by modem; feedback from diabetes nurse. n.b. usual care was weekly voice calls	Y	1	HbA1c; Provider time	NS diff in HbA1c in T/M group compared to control; NS diff in provider time	NONE
Biermann 2002	43	Intensive insulin therapy	BG meter transmitted values via modem every 1–3 weeks; physician phoned back if necessary	Y	8	HbA1c	NS difference between T/M and control groups (although HbA1c fell significantly in both)	NONE
Billiard 1991	22	IDDM for >2 y	BG data transmitted to central database	Y	3	HbA1c	Sig fall in HbA1c in the T/M period and not in the control period	POSITIVE
Bond 2007 & Bond 2010	62	Diabetes for at least 1 year	We-based system for patient to record BG, exercise, weight, BP, medication. Nurse contact via email or online chat. Weekly online discussion groups	Y	6	HbA1c; Weight; BP; HDL; Cholesterol; Depression; Social support; QoL; Self-efficacy	Sig improvements in all outcomes in T/M group relative to control	POSITIVE
Chase 2003	70 (63 completed the study)	Type 1 for at least a year	Transmission of BG data via modem every 2 weeks	Y	6	HbA1c; Cost of care	NS difference in HbA1c between T/M and control groups (although HbA1c fell significantly in both); Sig lower cost of care in T/M group	MILDLY POSITIVE
Dale 2009	231	Raised HbA1c	Telephone support from nurse specialist or from a peer-supporter (two arms in trial). Five calls on average.	N	6	Self-efficacy; HbA1c	No differences between groups for self-efficacy; No differences for other outcomes	NONE
Fallucca 1996	19	IDDM pregnant	Patient transmitted BG, exercise, urine data, plus exercise and insulin, each week. Recommendations received from specialist	Y	5	Blood glucose	BG sig lower in T/M group at end of study than in controls	POSITIVE
Gay 2006	100	Children and adolescents with Type 1	Every 2 weeks child went to pharmacist who downloaded the glucometer data and faxed it to the hospital; doctor responded within 5 days	Y	6	HbA1c	NS difference	NONE
Gomez 2002	10	Type 1	BG data transmitted every 2 weeks; web site for messages from pt and from doctor n.b. this was a crossover RCT	Y	6 (plus 6 months as control)	HbA1c; Hypoglycaemic episodes; QoL	NS diff in HbA1c in T/M group compared to control	NONE
Harno 2006	175	Type 1 and Type 2	Transmission of BG data via modem	Y	12	HbA1c; BP; biochem; Visits	Sig lower HbA1c in T/M group; dBP and some biochem sig lower; Fewer visits to doctor/nurse	POSITIVE
Horan 1990	20	IDDM for at least 1 year	Insulin and BG data sent by post each week. Home computer for plotting data and modules for self-education	Y	4.5	HbA1c; BG; Frequency of testing; Diabetes knowledge	NS diff in HbA1c; BG levels at pre-lunch and dinner sig better in T/M group than in control; Frequency of testing higher; NS difference in diabetes knowledge	MILDLY POSITIVE

Howe 2005	75	Children with Type 1	Telephone case management	No	6	HbA1c; Diabetes knowledge; Parent-child teamwork; Adherence	NS diff in HbA1c; NS diff in knowledge; Sig improvement in teamwork; Sig improvement in adherence	MILDLY POSITIVE
Izquierdo 2009	41	School children with Type 1	Monthly videoconference with school nurse and patient, and specialist diabetes nurse. Graphical/tabular data could be exchanged.	Y	6	HbA1c	Sig improvement in HbA1c in T/M group compared with controls.	POSITIVE
Jansa 2006	40	Type 1 with poor control	Transmission of BG data via modem approx every 2 weeks	Y	6	HbA1c; Hypoglycaemic episodes; Self-management; QoL; Cost	NS difference between T/M and control groups (although HbA1c fell significantly in both); Lower (but not sig?) costs in T/M group	MILDLY POSITIVE
Kim 2003	50	Diabetes (Type 2?)	Telephone contact with with diabetes nurse for insulin adjustment; average 16 calls per 12 weeks	Y (sort of)	3	HbA1c; Adherence	NS diff in HbA1c in T/M group compared to control; Sig better exercise adherence	MILDLY POSITIVE
Kwon 2004	110	Type 2 for at least 1 year	Web site for pt to store BG data and doctor to provide recommendations	Y	3	HbA1c	Sig lower HbA1c in T/M group	POSITIVE
Ladyzynski 2007 & Wojcicki 2001	30	Type 1, pregnant	Transmission of BG data via modem daily	Y	6	HbA1c; Variability in HbA1c	NS diff in HbA1c; Sig less variability	MILDLY POSITIVE
Lavery 2004	85	High-risk diabetes	Patients measured skin temp at 6 sites on feet twice a day; if temp diffs, they contacted the nurse	Y (sort of)	6	Number of diabetic foot complications; QoL	Sig fewer foot complications in T/M group compared to controls; NS diff in QoL	POSITIVE
Lawson 2005	46	Adolescents with type 1	Weekly telephone calls from diabetes nurse	Yes	6	HbA1c; Compliance; QoL	NS diffs in outcomes between groups; Sig improvementin HbA1c 6 months afterwards	MILDLY POSITIVE
Malijanian 2005	507	Type 1 or Type 2	Weekly educational phone calls for 12 weeks	N	12	HbA1c; QoL; annual eye exam; physician foot exam	NS diff in HbA1c in T/M group compared to control; Sig better adherence to ADA standards of eye exams, foot exams etc	MILDLY POSITIVE
Marrero 1995	106	Paediatric pts, diabetes	BG data transmitted every 2 weeks by modem; feedback from diabetes nurse	Y	12	HbA1c; Hospitalizations; ER visits; QoL; Nurse time	NS diff in HbA1c in T/M group compared to control; NS diff in hospitalizations, ER visits; Sig less nurse time	MILDLY POSITIVE
McKay 2001	78	Type 2	Personalised activity coaching plus online support from coach	Y	2	Physical activity; Depression	NS diff in outcomes between the groups	NONE
McMahon 2005	104	HbA1c $\geq$ 9%	Automatic transmission of BG and BP data to web site. Care manager provided recommendations via web site	Y	12	HbA1c; BP; Biochem	Sig lower HbA1c in T/M group; Sig lower sBP in T/M group; Sig improvements in some biochem	POSITIVE
Montori 2004	31	Type 1	BG data transmitted every 2 weeks by modem; feedback from diabetes nurse	Y	6	HbA1c	Sig lower HbA1c in T/M group compared to control	POSITIVE
Nunn 2006	123	Adolescents with type 1	Phone calls every 2 weeks with educator	Y	7	HbA1c; Admission rates; Diabetes knowledge; Compliance	NS differences	NONE
Panagiotopoulos 2003	50	Adolescents with type 1	Weekly telephone calls from nurse educator. Nurse recorded BG values and made insulin adjustments	Yes	6	HbA1c; Insulin dosage	NS diff between groups in change in HbA1c; Sig improvement in T/M subset with high HbA1c; No diff in insulin	MILDLY POSITIVE
Piette 2000	248	Diabetes	IVR "bi-weekly" calls for education and reporting BG readings using the phone	Y	12	Depression; QoL; Self-efficacy; Days in bed	Sig less depression in T/M group compared to control; No effect on anxiety or QoL; Sig better self-efficacy; Sig fewer days in bed	MILDLY POSITIVE

(Continued)

Table 5 Continued

Study	No of subjects	Type of pt	Intervention	Telemonitoring?	Duration (months)	Outcomes	Result	Overall value of intervention (on HbA1c)
Piette 2001	272	Diabetes	Telephone – IVR (approx monthly) and nurse follow-up calls	Y	12	HbA1c; Podiatry visits	Sig lower HbA1c in T/M group for subgroups with high HbA1c at baseline; More podiatry visits	MIILDLY POSITIVE
Quinn 2008	30	Type 2	BG data transmitted immediately. Educational material sent to mobile phone daily	Y	3	HbA1c; Satisfaction	Sig greater reduction in HbA1c in T/M group than control; Improved satisfaction	POSITIVE
Ralston 2009	83	Type 2	Web-based care management. Weekly upload of BG readings. Email dialogue with care manager	Y	12	HbA1c	Sig reduction in HbA1c in T/M group compared to control group	POSITIVE
Rami 2006	36	Adolescents with Type 1	Cross-over RCT. BG data uploaded via SMS. Weekly feedback about glycaemic control	Y	3	HbA1c	Sig improvement in HbA1c during T/M phase of crossover	POSITIVE
Shea 2006 & Shea 2009	1665	Diabetes	Home T/M unit with automatic transmission of BG and BP data, and videoconferencing	Y	12	HbA1c; BP; LDL	Sig lower HbA1c in T/M group compared to control; Sig lower BP; Sig lower cholesterol	POSITIVE [Same results at 5 years]
Shultz 1992	20	Diabetes	BG data transmitted weekly. Clinician used the computer generated report during OPD visits n.b. this was a crossover RCT	Y	6 (plus 9 months as control)	HbA1c	Sig lower HbA1c in T/M group compared to control	POSITIVE
Thomson 1999	46	Poorly controlled diabetes	Telephone contact with diabetes nurse for insulin adjustment; average 3 calls per week	N	6	HbA1c	Sig lower HbA1c in T/M group compared to control	POSITIVE
Tsang 2001	20	Diabetes	Electronic diary to record food items; automatic transmission by modem every 2 days. n.b. this was a crossover RCT	Y	3 (plus 3 months control period)	HbA1c	Sig lower HbA1c in T/M group compared to control	POSITIVE
Whitlock 2000	28	Diabetes	Home T/M unit – BP and videoconferencing; weekly visits	Y	3	HbA1c; Bodyweight	Sig lower HbA1c in T/M group compared to control	POSITIVE
Wong 2005	101	Type 1 or Type 2 needing hospitalization for glycaemic control	Telephone contact with diabetes nurse for insulin adjustment; average 1 call per 1-2 week	N	6	HbA1c; Self care; Health care utilization; Satisfaction	NS diff in HbA1c in T/M group compared to control; Sig better exercise adherence	MILDLY POSITIVE

Diabetes RCTs

Ahring KK, Ahring JP, Joyce C, Farid NR. Telephone modem access improves diabetes control in those with insulin-requiring diabetes. *Diabetes Care* 1992;**15**:971–975

Bellazzi R, Arcelloni M, Bensa G, *et al.* Design, methods, and evaluation directions of a multi-access service for the management of diabetes mellitus patients. *Diabetes Technol Ther* 2003;**5**:621–629

Bergenstal RM, Anderson RL, Bina DM, *et al.* Impact of modem-transferred blood glucose data on clinician work efficiency and patient glycemic control. *Diabetes Technol Ther* 2005;**7**:241–247

Biermann E, Dietrich W, Rihl J, Standl E. Are there time and cost savings by using telemanagement for patients on intensified insulin therapy? A randomised, controlled trial. *Comput Methods Programs Biomed* 2002;**69**:137–146

Billiard A, Rohmer V, Roques MA, *et al.* Telematic transmission of computerized blood glucose profiles for IDDM patients. *Diabetes Care* 1991;**14**:130–134

Bond GE, Burr R, Wolf FM, Price M, McCurry SM, Teri L. The effects of a web-based intervention on the physical outcomes associated with diabetes among adults age 60 and older: a randomized trial. *Diabetes Technol Ther* 2007;**9**:52–59

Bond GE, Burr RL, Wolf FM, Feldt K. The effects of a web-based intervention on psychosocial well-being among adults aged 60 and older with diabetes: a randomized trial. *Diabetes Educ* 2010;**36**:446–456

Chase HP, Pearson JA, Wightman C, Roberts MD, Oderberg AD, Garg SK. Modem transmission of glucose values reduces the costs and need for clinic visits. *Diabetes Care* 2003;**26**:1475–1479

Dale J, Caramlau I, Sturt J, Friede T, Walker R. Telephone peer-delivered intervention for diabetes motivation and support: the telecare exploratory RCT. *Patient Educ Couns* 2009;**75**:91–98

Fallucca F. Telemedicine in the treatment of diabetic pregnancy *Practical Diabetes International* 1996;**13 (4)**:115–118

Gay CL, Chapuis F, Bendelac N, Tixier F, Treppoz S, Nicolino M. Reinforced follow-up for children and adolescents with type 1 diabetes and inadequate glycaemic control: a randomized controlled trial intervention via the local pharmacist and telecare. *Diabetes Metab* 2006;**32**:159–165

Gómez EJ, Hernando ME, García A, *et al.* Telemedicine as a tool for intensive management of diabetes: the DIABTel experience. *Comput Methods Programs Biomed* 2002;**69**:163–177

Harno K, Kauppinen-Mäkelin R, Syrjäläinen J. Managing diabetes care using an integrated regional e-health approach. *J Telemed Telecare* 2006;**12** (Suppl. 1): 13–15

Horan PP, Yarborough MC, Besigel G, Carlson DR. Computer-assisted self-control of diabetes by adolescents. *Diabetes Educ* 1990;**16**:205–211

Howe CJ, Jawad AF, Tuttle AK, *et al.* Education and telephone case management for children with type 1 diabetes: a randomized controlled trial. *J Pediatr Nurs* 2005;**20**:83–95

Izquierdo R, Morin PC, Bratt K, *et al.* School-centered telemedicine for children with type 1 diabetes mellitus. *J Pediatr* 2009;**155**:374–379

Jansa M, Vidal M, Viaplana J, *et al.* Telecare in a structured therapeutic education programme addressed to patients with type 1 diabetes and poor metabolic control. *Diabetes Res Clin Pract* 2006;**74**:26–32

Kim H-S, Oh J-A. Adherence to diabetes control recommendations: impact of nurse telephone calls. *J Adv Nurs* 2003;**44**:256–261

Kwon H-S, Cho J-H, Kim H-S, *et al.* Establishment of blood glucose monitoring system using the internet. *Diabetes Care* 2004;**27**:478–483
Ładyzynski P, Wójcicki JM. Home telecare during intensive insulin treatment–metabolic control does not improve as much as expected. *J Telemed Telecare* 2007;**13**:44–47
Lavery LA, Higgins KR, Lanctot DR, *et al.* Home monitoring of foot skin temperatures to prevent ulceration. *Diabetes Care* 2004;**27**:2642–2647
Lawson ML, Cohen N, Richardson C, Orrbine E, Pham B. A randomized trial of regular standardized telephone contact by a diabetes nurse educator in adolescents with poor diabetes control. *Pediatr Diabetes* 2005;**6**:32–40
Maljanian R, Grey N, Staff I, Conroy L. Intensive telephone follow-up to a hospital-based disease management model for patients with diabetes mellitus. *Dis Manag* 2005;**8**:15–25
Marrero DG, Vandagriff JL, Kronz K, *et al.* Using telecommunication technology to manage children with diabetes: the Computer-Linked Outpatient Clinic (CLOC) Study. *Diabetes Educ* 1995;**21**:313–319
McKay HG, King D, Eakin EG, Seeley JR, Glasgow RE. The diabetes network internet-based physical activity intervention: a randomized pilot study. *Diabetes Care* 2001;**24**:1328–1334
McMahon GT, Gomes HE, Hohne SH, Hu TM-J, Levine BA, Conlin PR. Web-based care management in patients with poorly controlled diabetes. *Diabetes Care* 2005;**28**:1624–1629
Montori VM, Helgemoe PK, Guyatt GH, *et al.* Telecare for patients with type 1 diabetes and inadequate glycemic control: a randomized controlled trial and meta-analysis. *Diabetes Care* 2004;**27**:1088–1094
Nunn E, King B, Smart C, Anderson D. A randomized controlled trial of telephone calls to young patients with poorly controlled type 1 diabetes. *Pediatr Diabetes* 2006;**7**:254–259
Panagiotopoulos C. Weekly telephone contact by a diabetes educator in adolescents with type 1 diabetes *Canadian Journal of Diabetes* 2003;**27**:422–427
Piette JD, Weinberger M, Kraemer FB, McPhee SJ. Impact of automated calls with nurse follow-up on diabetes treatment outcomes in a Department of Veterans Affairs Health Care System: a randomized controlled trial. *Diabetes Care* 2001;**24**:202–208
Piette JD, Weinberger M, McPhee SJ. The effect of automated calls with telephone nurse follow-up on patient-centered outcomes of diabetes care: a randomized, controlled trial. *Med Care* 2000;**38**:218–230
Quinn CC, Clough SS, Minor JM, Lender D, Okafor MC, Gruber-Baldini A. WellDoc mobile diabetes management randomized controlled trial: change in clinical and behavioral outcomes and patient and physician satisfaction. *Diabetes Technol Ther* 2008;**10**:160–168
Ralston JD, Hirsch IB, Hoath J, Mullen M, Cheadle A, Goldberg HI. Web-based collaborative care for type 2 diabetes: a pilot randomized trial. *Diabetes Care* 2009;**32**:234–239
Rami B, Popow C, Horn W, Waldhoer T, Schober E. Telemedical support to improve glycemic control in adolescents with type 1 diabetes mellitus. *Eur J Pediatr* 2006;**165**:701–705
Shea S, Weinstock RS, Starren J, *et al.* A randomized trial comparing telemedicine case management with usual care in older, ethnically diverse, medically underserved patients with diabetes mellitus. *J Am Med Inform Assoc* 2006;**13**:40–51
Shea S, Weinstock RS, Teresi JA, *et al.* A randomized trial comparing telemedicine case management with usual care in older, ethnically diverse, medically underserved patients with diabetes mellitus: 5 year results of the IDEATel study. *J Am Med Inform Assoc* 2009;**16**:446–456
Shultz EK, Bauman A, Hayward M, Holzman R. Improved care of patients with diabetes through telecommunications. *Ann N Y Acad Sci* 1992;**670**:141–145
Thompson DM, Kozak SE, Sheps S. Insulin adjustment by a diabetes nurse educator improves glucose control in insulin-requiring diabetic patients: a randomized trial. *CMAJ* 1999;**161**:959–962
Tsang MW, Mok M, Kam G, *et al.* Improvement in diabetes control with a monitoring system based on a hand-held, touch-screen electronic diary. *J Telemed Telecare* 2001;**7**:47–50
Whitlock WL, Brown A, Moore K, *et al.* Telemedicine improved diabetic management. *Mil Med* 2000;**165**:579–584
Wojcicki JM, Ladyzynski P, Krzymien J, *et al.* What we can really expect from telemedicine in intensive diabetes treatment: results from 3-year study on type 1 pregnant diabetic women. *Diabetes Technol Ther* 2001;**3**:581–589
Wong FKY, Mok MPH, Chan T, Tsang MW. Nurse follow-up of patients with diabetes: randomized controlled trial. *J Adv Nurs* 2005;**50**:391–402

Table 6 Heart failure RCTs

Study	No of subjects	Type of pt	Intervention	Telemonitoring?	Duration (months)	Outcomes	Result	Overall value of intervention
Abraham 2011	550	NYHA 3	Wireless implantable pulmonary artery monitoring; daily measurements	Y	15	Hospitalization; Mortality; QoL	Sig lower hospitalization in T/M group than control; Sig lower admission/mortality; Sig better QoL	POSITIVE
Antonicelli 2010 & Antonicelli 2008	57	NYHA 2–4	Weekly phone contact for symptoms, adherence, and previous day's BP, HR, wt and urine output. Weekly ECG transmission	Y	12	Mortality combined with Readmission	Sig lower mortality/readmission in T/M group	POSITIVE
Artinian 2003	18	NYHA 2–4	Home monitoring for medication, symptoms, BP and weight. Automatic upload. Clinicians had web interface	Y	3	Self-care behaviour; QoL; Functional status	NS difference in self-care behaviour or functional status between the groups; Sig improvement in QoL in T/M group	MILDLY POSITIVE
Balk 2008	214	NYHA 1–4	Educational TV	N	9	Days in hospital; Mortality	No differences	NONE
Barnason 2003	35	NYHA 1–2 & CABG	Daily questions (Health Buddy) on symptoms etc plus education	Y	1.5	Self-efficacy; CAD risk; Functioning	Sig better self-efficacy in T/M group ?NS diff in CAD risk; Sig better functioning	POSITIVE
Barth 2001	34	CHF patients at discharge	Telephone calls from case manager approx every 2-weeks	Y (symptoms not vital signs data)	2	Unexpected office visits; ER visits; Readmissions; QoL	NS diff in first 3 outcomes; Sig better QoL in T/M group compared to controls	MILDLY POSITIVE
Benetar, 2003	216	NYHA 3 or 4	Daily bodyweight, BP, HR, oxygen saturation	Y	3	Hospitalizations; Costs; QoL	Fewer hospitalizations; Lower costs; Improved QoL	POSITIVE
Bourge 2008	274	NYHA 3	Wireless implantable pulmonary artery monitoring;**weekly** measurements	Y	6	HF-related event (hospitalization, ED visit); Time to hospitalization	No diff in HF-related events; Sig better time to hospitalization in T/M group compared to control	MILDLY POSITIVE

(Continued)

Table 6 Continued

Study	No of subjects	Type of pt	Intervention	Telemonitoring?	Duration (months)	Outcomes	Result	Overall value of intervention
Cabezas 2006	134	NYHA 1-4	Information provided to patient and then telephone calls from pharmacist (monthly for first 6 months, then every 2 months)	N	12	Time to first hospital re-admission; Number of re-admissions; Hospital stay	Fewer re-admissions in the T/M group and shorter hospital stay – sig at 6 months, but not at 12 months	MILDLY POSITIVE
Capomolla 2004	133	NYHA 2-4	Vital signs and symptoms data sent by IVR system.	Y	12	Hospital admissions; ER use; Mortality	Sig lower Hosp admission and ER use in T/M group compared with controls; NS diff in mortality	POSITIVE
Chaudhry, 2010	1653	NYHA 2 or 3 (mainly)	Daily bodyweight and symptoms collected by IVR	Y (sort of)	6	Hospitalizations; Mortality	NS change in admissions; NS change in mortality	NONE
Cleland, 2005	426	Most had NYHA 4 episode(s)	Twice daily bodyweight, BP, HR, ECG (also had a second arm with nurse-telephone support)	Y	8	Hospitalizations; Mortality	Lower mortality and fewer hospitalizations	POSITIVE
Copeland 2010	458	CHF	Nurse advice line (telephone)	N	12	Hospital stay; Re-admissions; QoL; Costs	No differences between groups in clinical outcomes; Costs were significantly higher in the T/M group	WEAKLY NEGATIVE
Dansky, 2008	284	Heart failure	Daily bodyweight, pulse, BP. Also had a second arm with video and stethoscope as well as monitoring	Y	4	Hospitalizations; ED visits; Mortality	Fewer hospitalizations and fewer ED visits in T/M group, but NS; also NS difference in mortality; Video better than monitoring alone	NONE
Dansky 2009	108	CHF	Health Buddy to collect symptoms and compliance data	Y	6	Admissions; ED visits	Sig lower hospital admissions and ED visits in T/M group	POSITIVE
Dar 2009	182	NYHA 2–4	Daily transmission of weight, BP, HR and O2 sat to specialist nurse	Y	6	Non-elective hospitalizations; HR-related admissions; QoL; Costs	NS diff in primary outcome; NS diff in secondary outcomes	NONE
DeBusk 2004	462	NYHA 1–4	Telephone calls from nurse manager (n = 16)	N	12	Time to rehospitalization; Time to composite outcome; Medication	NS diffs	NONE
DeWalt 2006	123	NYHA 2–4	Telephone calls (n = 10) from educator	N	6	Mortality/Re-admission; QoL	Sig lower rate of hospitalization or death in T/M group; NS diff in QoL	POSITIVE
Domingo 2011	105	NYHA 2–3	All pts had educational videos, questionnaires etc. Intervention was daily self-monitoring of weight, HR and BP. Data sent automatically to server.	Y	12	Behaviour change	Sig improvement in behaviour in T/M group compared to control	POSITIVE
Dougherty 2005	168	Cardiac arrest survivors, with ICD	Weekly telephone education	N	2	Physical functioning; Psychological adjustment; Health care utilization	Sig better physical functioning (symptoms and concerns) in T/M group; Sig better psychological adjustment; NS diffs in utilization	POSITIVE
Dunagan 2005	151	NYHA 2–4	Weekly nurse telephone calls for education and monitoring	N	12	Time to hospital encounter (admission or ED visit); Mortality	Sig longer time to hospital encounter in T/M group; NS diffs in mortality	POSITIVE
Ferrante 2010 & GESICA 2005	1518	NYHA 3–4	Nurse telephone calls every 2 weeks for education, compliance etc	N	12	Mortality; Hospital admission for HF	Sig lower mortality in T/M group (confirmed at 3 year follow-up); Sig fewer hospital admissions	POSITIVE
Galbreath 2004	1069	NYHA 1–4	Telephone-administered disease management; weekly then monthly calls. [n.b. half of the intervention group had home monitors, but "data not used in clinical management"]	N	18	All-cause mortality; Walk test; NYHA class; Costs	Sig reduction in mortality in T/M group; Walk test NS; NYHA class sig better; Costs NS different	POSITIVE
Gattis 1999	181	NYHA 1–4	Pharmacist discussed patient's case and provided 3 telephone follow up calls	N	6	All-cause mortality; HF clinical events; Medication use	All-cause mortality and HF events sig lower in T/M group than in controls; Medication use sig higher in T/M group	POSITIVE

Giordana, 2009	460	NYHA 2–4	ECG transmission via PSTN on demand; call centre nurse contact every week	Y (sort of)	12	Hospitalizations; Mortality; Costs	Fewer admissions; lower costs; NS change in mortality	POSITIVE
Goldberg, 2003	280	NYHA 3 or 4	Twice daily bodyweight and symptom questions via home device	Y	6	Hospitalizations; ED visits; Mortality	No difference in hospitalization or ED visits; Improved survival	MILDLY POSITIVE
Jerant 2003 & Jerant 2001	37	NYHA 2–4	T/M group 1 had telephone contact T/M group 2 had home video contact and electronic stethoscope	N	2	CHF-related cost of readmission; Cost of ED visits	NS difference in cost of re-admissions in T/M group compared to control; Sig lower cost of ED visits	MILDLY POSITIVE
Kashem 2008 & Kashem 2006	48	NYHA 2–4	Weekly data transmission of BP, HR, weight. Web messaging.	Y	12	ED visits; Hospital admissions; Hospital stay	Sig fewer ED visits in the T/M group; Sig fewer admissions and lower stay	POSITIVE
Kielblock 2007	502	NYHA 1–4	Daily bodyweight sent by modem or SMS. Counselling by phone every 2 weeks for 3 months	Y	12	Hospital stay; Cost; Mortality	Sig shorter hospital stay in T/M group than control; Sig lower cost; Sig reduced mortality	POSITIVE
Koehler 2011	710	NYHA 2–3	Daily ECG, BP, weight sent automatically via home PDA	Y	26	Mortality; Hospitalization for HF	NS diff in mortality or hospitalization	NONE
Konstam 2011	88	Recent admission for HF	Daily bodyweight, vital signs, clinical status data sent automatically to nurse manager	Y	3	QoL	NS diff in QoL between T/M and control groups	NONE
Kulshreshtha 2010	150	HF, high-risk of readmission	Daily transmission of weight, BP, PR, oximetry. Symptom-related questions via home device	Y	6	Readmission	NS difference in readmission rates	NONE
Laramee 2003	287	NYHA 1–4	Approx weekly telephone calls from case manager for 12 weeks. Symptoms were discussed	Y (sort of)	3	All-cause readmission; Adherence; Satisfaction; Medication dosage; Cost	NS difference in readmissions; Adherence, satisfaction, medications sig better in T/M group than controls; NS difference in costs	MILDLY POSITIVE
Leventhal 2011	42	NYHA 2–4	Nurse home visit plus 17 phone calls over a year	Y (sort of)	12	Mortality; Readmission; QoL	NS differences	NONE
de Lusignan 1999	20	NYHA 1–4	Daily BP, PR and weight sent to server automatically; weekly videoconference with nurse	Y	3	BP; weight; QoL	QoL improved significantly in the T/M group; no change in BP, weight	MILDLY POSITIVE
de Lusignan 2001	20	NYHA 1–4	Automatic transmission of vital signs. Videoconferences	Y	12	QoL	NS differences in QoL between T/M and control group	NONE
Mortara, 2009	461	NYHA 2–4	Three interventions (monthly nurse phone call; plus weekly vital signs via IVR system; plus 24-h recording of ECG, respiration, activity) - but lumped into one home telemed group	Y (sort of)	12	Hospitalizations; Mortality	NS change in admissions; NS change in mortality	NONE
Piotrowicz 2010	152	NYHA 2–3	Patients answered phone Qs about symptoms etc at the start of home rehab sessions, then transmitted ECGs before and after home exercise	Y	2	Adherence; QoL	Adherence was better in the T/M group; QoL was the same	MILDLY POSITIVE
Rainville 1999	34	NYHA 2–4	Pharmacist review; five phone calls to patients	Y (sort of)	12	Readmissions for HF; Mortality; Time to readmission	Readmissions for HF sig lower in T/M group than control; Death/ readmission sig lower; Time to readmission sig better in T/M group	POSITIVE
Ramachandran 2007	50	NYHA 1–4	Weekly phone calls from junior doctors; telephone help line	Y (sort of)	6	QoL; Functional capacity	QoL sig better in T/M group than control; Funcional capacity sig better in T/M group	POSITIVE
Ramaekers 2009	101	Heart failure	Home device (Health Buddy) sent data on symptoms, health behaviour and disease knowledge) to server	Y	3	Disease-specific knowledge; Adherence; Depression	Disease knowledge sig better in T/M group than control in 2 of the 3 regions; Adherence – mainly similar Depression – NS diff	MILDLY POSITIVE
Riegel 2006	135	NYHA 2–4	Telephone management by nurse; phone call every 2 weeks	Y (sort of)	6	HF hospitalization; Readmissions; Days in hospital; Cost	NS differences	NONE
Riegel 2002	242	NYHA 2–4	Telephone management by nurse; phone call every 2 weeks	Y (sort of)	6	Hospitalization rate; Resource utilization; Cost	Sig lower hospitalization rate in T/M group; Sig lower utilization and cost	POSITIVE
Ross 2004	107	NYHA 2–3	Web-based patient-accessible electronic record, educational guide and messaging system	N	12 months	Self efficacy; Health status; Adherence	NS differences	NONE

(Continued)

Table 6 Continued

Study	No of subjects	Type of pt	Intervention	Telemonitoring?	Duration (months)	Outcomes	Result	Overall value of intervention
Scherr 2009	120	NYHA 2–4	Daily transmission of BP, HR, weight and medication	Y	6	Cardiovascular mortality or rehospitalization; Functional status; Length of hospital stay	NS reduction in mortality/hospitalizations in T/M group; Sig shorter length of stay in hospital	MILDLY POSITIVE
Schwarz 2008	102	NYHA 2–4	Daily transmission of weight and symptoms (questions via home device)	Y	3	Hospital admissions; ED visits; Cost of care; Depressive symptoms; QoL	No sig diffs between groups	NONE
Sisk 2006 & Hebert 2008	406	NYHA 1–4	Telephone calls from nurse manager	N	12	Hospitalizations; Self-reported functioning; Cost; QALY	Sig fewer hospitalizations and better functioning in T/M group compared to controls; Cost higher in T/M group, but so also was QoL – intervention probably cost-effective for less severe HF	POSITIVE
Smith 2008 (n.b. reanalysis of data from Galbreath 2004)	1069	NYHA 1–4	Telephone management by nurse, weekly then monthly calls; half of the intervention group also had home monitoring (BP, oximeter)	Y	17	Mortality; Clinical outcomes; Cost	Sig reduced mortality in T/M group; Some clinical outcomes better; No diff in costs	POSITIVE
Soran, 2008	315	LVEF < 40% (most NYHA 2 or 3)	Daily bodyweight and symptom questions via home device	Y	6	CV mortality/ Hospitalizations	NS difference in hospitalizations or mortality	NONE
Spaeder 2006	49	NYHA 2–3	IVR system used to collect daily weight, PR, BP	Y	3	Titration time (time from starting carvedilol to achieving final dose)	Sig faster to achieve final dose in T/M group	POSITIVE
Tompkins, 2010	390	Heart failure	Home monitor for daily bodyweight, BP, HR, blood oxygen	Y	6	Hospital days; ED visits; Costs	Sig fewer hospital days; Fewer ED visits (NS); More urgent care visits; Lower costs	POSITIVE
Tsuyuki 2004	276	NYHA 1–4	Telephone contact by educator for 6 months	N	6	Medication adherence; Clinical events	NS diff in adherence between T/M group and control; Sig lower ED visits and hospital stay in T/M group	MILDLY POSITIVE
Wade 2011	316	High risk HF	Transmission of weight, BP 5 times per week; questions on symptoms, activities periodically	Y	6	Hospitalization; ED visit; Mortality	NS differences	NONE
Wakefield 2009 & Wakefield 2008	148	NYHA 2–4	Two arms. Gp 1 had weekly telephone contact with nurse manager. Gp 2 had home videophone for nurse contacts	Y	12	Hospital readmission; Mortality; QoL	Fewer admissions in T/M group than in controls, but ?NS; combined T/M groups were sig better; NS diff in mortality or QoL	MILDLY POSITIVE
Weintraub 2010	188	NYHA 1–4	Home device for daily transmission of body weight, BP, HR	Y	3	Hospitalization for HF; Mortality; Hospital days	Sig fewer hospitalizations in T/M group; NS diff in mortality	POSITIVE
Woodend 2008	121	NYHA 2–4	Daily transmission of weight and BP; periodic transmission of ECG; weekly videoconferences [Excluding the angina patients in the study . . . HF data only]	Y	3	Readmissions; Hospital stay	NS diffs at 3 months. Sig fewer hospital admission in T/M group after 1 year	MILDLY POSITIVE
Wootton 2009	409	CHF	Telephone contact with care coordinator	N	12	Cost QoL	NS diff in costs; NS diff in QoL	NONE

Heart failure RCTs
Abraham WT, Adamson PB, Bourge RC, *et al.* Wireless pulmonary artery haemodynamic monitoring in chronic heart failure: a randomised controlled trial. *Lancet* 2011;**377**:658–666
Antonicelli R, Mazzanti I, Abbatecola AM, Parati G Impact of home patient telemonitoring on use of beta-blockers in congestive heart failure. *Drugs Aging* 2010;**27**:801–805
Antonicelli R, Testarmata P, Spazzafumo L, *et al.* Impact of telemonitoring at home on the management of elderly patients with congestive heart failure. *J Telemed Telecare* 2008;**14**:300–305
Artinian NT, Harden JK, Kronenberg MW, *et al.* Pilot study of a Web-based compliance monitoring device for patients with congestive heart failure. *Heart Lung* 2003;**32**:226–233
Balk AH, Davidse W, van Dommelen P, *et al.* Tele-guidance of chronic heart failure patients enhances knowledge about the disease. A multi-centre, randomised controlled study. *Eur J Heart Fail* 2008;**10**:1136–1142
Barnason S, Zimmerman L, Nieveen J, Schmaderer M, Carranza B, Reilly S. Impact of a home communication intervention for coronary artery bypass graft patients with ischemic heart failure on self-efficacy, coronary disease risk factor modification, and functioning. *Heart Lung* 2003;**32**:147–158

Barth V. A nurse-managed discharge program for congestive heart failurepatients: outcomes and costs *Home Health Care Management and Practice* 2001;**13**:436–443
Benatar D, Bondmass M, Ghitelman J, Avitall B. Outcomes of chronic heart failure. *Arch Intern Med* 2003;**163**:347–352
Bourge RC, Abraham WT, Adamson PB, *et al.* Randomized controlled trial of an implantable continuous hemodynamic monitor in patients with advanced heart failure: the COMPASS-HF study. *J Am Coll Cardiol* 2008;**51**:1073–1079
Cabezas CL, Salvador CF, Quadrada DC, *et al.* Randomized clinical trial of a postdischarge pharmaceutical care program vs regular follow-up in patients with heart failure. *Farm Hosp* 2006;**30**:328–342
Capomolla S, Pinna G, La Rovere MT, *et al.* Heart failure case disease management program: a pilot study of home telemonitoring versus usual care. *Eur Heart J* 2004;**6** (Suppl. F): F91–F98
Chaudhry SI, Barton B, Mattera J, Spertus J, Krumholz, HM. Randomized trial of telemonitoring to improve heart failure outcomes (Tele-HF): study design. *J Card Fail* 2007;**13**:709–714
Cleland JG, Louis AA, Rigby AS, *et al.* Noninvasive home telemonitoring for patients with heart failure at high risk of recurrent admission and death: the Trans-European Network-Home-Care Management System (TEN-HMS) study. *J Am Coll Cardiol* 2005;**45**:1654–1664
Copeland LA, Berg GD, Johnson DM, Bauer RL. An intervention for VA patients with congestive heart failure. *Am J Manag Care* 2010;**16**:158–165
Dansky K, Vasey J. Managing heart failure patients after formal homecare. *Telemed J E Health* 2009;**15**:983–991
Dansky KH, Vasey J, Bowles K. Impact of telehealth on clinical outcomes in patients with heart failure. *Clin Nurs Res* 2008;**17**:182–199
Dar O, Riley J, Chapman C, *et al.* A randomized trial of home telemonitoring in a typical elderly heart failure population in North West London: results of the Home-HF study. *Eur J Heart Fail* 2009;**11**:319–325
DeBusk RF, Miller NH, Parker KM, *et al.* Care management for low-risk patients with heart failure: a randomized, controlled trial. *Ann Intern Med* 2004;**141**:606–613
DeWalt DA, Malone RM, Bryant ME, *et al.* A heart failure self-management program for patients of all literacy levels: a randomized, controlled trial [ISRCTN11535170]. *BMC Health Serv Res* 2006;**6**:30
Domingo M, Lupón J, González B, *et al.* Evaluation of a telemedicine system for heart failure patients: feasibility, acceptance rate, satisfaction and changes in patient behavior. Results from the CARME (CAtalan Remote Management Evaluation) study. *Eur J Cardiovasc Nurs* 2011; Mar 12 [Epub ahead of print]
Dougherty CM, Thompson EA, Lewis FM. Long-term outcomes of a telephone intervention after an ICD. *Pacing Clin Electrophysiol* 2005;**28**:1157–1167
Dunagan WC, Littenberg B, Ewald GA, *et al.* Randomized trial of a nurse-administered, telephone-based disease management program for patients with heart failure. *J Card Fail* 2005;**11**:358–365
Ferrante D, Varini S, Macchia A, *et al.* Long-term results after a telephone intervention in chronic heart failure: DIAL (Randomized Trial of Phone Intervention in Chronic Heart Failure) follow-up. *J Am Coll Cardiol* 2010;**56**:372–378
Galbreath AD, Krasuski RA, Smith B, *et al.* Long-term healthcare and cost outcomes of disease management in a large, randomized, community-based population with heart failure. *Circulation* 2004;**110**:3518–3526
Gattis WA, Hasselblad V, Whellan DJ, O'Connor CM. Reduction in heart failure events by the addition of a clinical pharmacist to the heart failure management team: results of the Pharmacist in Heart Failure Assessment Recommendation and Monitoring (PHARM) Study. *Arch Intern Med* 1999;**159**:1939–1945
GESICA Investigators. Randomised trial of telephone intervention in chronic heart failure: DIAL trial. *BMJ* 2005;**331**:425
Giordano A, Scalvini S, Zanelli E, *et al.* Multicenter randomised trial on home-based telemanagement to prevent hospital readmission of patients with chronic heart failure. *Int J Cardiol* 2009;**131**:192–199
Goldberg LR, Piette JD, Walsh MN, *et al.* Randomized trial of a daily electronic home monitoring system in patients with advanced heart failure: the Weight Monitoring in Heart Failure (WHARF) trial. *Am Heart J* 2003;**146**:705–712
Hebert PL, Sisk JE, Wang JJ, *et al.* Cost-effectiveness of nurse-led disease management for heart failure in an ethnically diverse urban community. *Ann Intern Med* 2008;**149**:540–548
Jerant AF, Azari R, Martinez C, Nesbitt TS. A randomized trial of telenursing to reduce hospitalization for heart failure: patient-centered outcomes and nursing indicators. *Home Health Care Serv Q* 2003;**22**:1–20
Jerant AF, Azari R, Nesbitt TS. Reducing the cost of frequent hospital admissions for congestive heart failure: a randomized trial of a home telecare intervention. *Med Care* 2001;**39**:1234–1245
Kashem A, Droogan MT, Santamore WP, Wald JW, Bove AA. Managing heart failure care using an internet-based telemedicine system. *J Card Fail* 2008;**14**:121–126
Kashem A, Droogan MT, Santamore WP, *et al.* Web-based Internet telemedicine management of patients with heart failure. *Telemed J E Health* 2006;**12**:439–447
Kielblock B, Frye C, Kottmair S, Hudler T, Siegmund-Schultze E, Middeke M. [Impact of telemetric management on overall treatment costs and mortality rate among patients with chronic heart failure]. *Dtsch Med Wochenschr* 2007;**132**:417–422 [German]
Koehler F, Winkler S, Schieber M, *et al.* Impact of remote telemedical management on mortality and hospitalizations in ambulatory patients with chronic heart failure: the telemedical interventional monitoring in heart failure study. *Circulation* 2011;**123**:1873–1880
Konstam V, Gregory D, Chen J, *et al.* Health-related quality of life in a multicenter randomized controlled comparison of telephonic disease management and automated home monitoring in patients recently hospitalized with heart failure: SPAN-CHF II trial. *J Card Fail* 2011;**17**:151–157
Kulshreshtha A, Kvedar JC, Goyal A, Halpern EF, Watson AJ. Use of remote monitoring to improve outcomes in patients with heart failure: a pilot trial. *Int J Telemed Appl* 2010;**2010**:870959
Laramee AS, Levinsky SK, Sargent J, Ross R, Callas P. Case management in a heterogeneous congestive heart failure population: a randomized controlled trial. *Arch Intern Med* 2003;**163**:809–817
Leventhal ME, Denhaerynck K, Brunner-La Rocca HP, *et al.* Swiss Interdisciplinary Management Programme for Heart Failure (SWIM-HF): a randomised controlled trial study of an outpatient inter-professional management programme for heart failure patients in Switzerland. *Swiss Med Wkly* 2011;**141**:w13171
de Lusignan S, Meredith K, Wells S, Leatham E, Johnson P. A controlled pilot study in the use of telemedicine in the community on the management of heart failure – a report of the first three months. *Stud Health Technol Inform* 1999;**64**:126–137
de Lusignan S, Wells S, Johnson P, Meredith K, Leatham E. Compliance and effectiveness of 1 year's home telemonitoring. The report of a pilot study of patients with chronic heart failure. *Eur J Heart Fail* 2001;**3**:723–730
Mortara A, Pinna GD, Johnson P, *et al.* Home telemonitoring in heart failure patients: the HHH study (Home or Hospital in Heart Failure). *Eur J Heart Fail* 2009;**11**:312–318
Piotrowicz E, Baranowski R, Bilinska M, *et al.* A new model of home-based telemonitored cardiac rehabilitation in patients with heart failure: effectiveness, quality of life, and adherence. *Eur J Heart Fail* 2010;**12**:164–171
Rainville EC. Impact of pharmacist interventions on hospital readmissions for heart failure. *Am J Health Syst Pharm* 1999;**56**:1339–1342
Ramachandran K, Husain N, Maikhuri R, *et al.* Impact of a comprehensive telephone-based disease management programme on quality-of-life in patients with heart failure. *Natl Med J India* 2007;**20**:67–73
Ramaekers BLT, Janssen-Boyne JJ, Gorgels APM, Vrijhoef HJM. Adherence among telemonitored patients with heart failure to pharmacological and nonpharmacological recommendations. *Telemed J E Health*;**15**:517–524
Riegel B, Carlson B, Glaser D, Romero T. Randomized controlled trial of telephone case management in Hispanics of Mexican origin with heart failure. *J Card Fail* 2006;**12**:211–219
Riegel B, Carlson B, Kopp Z, LePetri B, Glaser D, Unger A. Effect of a standardized nurse case-management telephone intervention on resource use in patients with chronic heart failure. *Arch Intern Med* 2002;**162**:705–712
Ross SE, Moore LA, Earnest MA, Wittevrongel L, Lin CT. Providing a web-based online medical record with electronic communication capabilities to patients with congestive heart failure: randomized trial. *J Med Internet Res* 2004;**6**:e12
Scherr D, Kastner P, Kollmann A, *et al.* Effect of home-based telemonitoring using mobile phone technology on the outcome of heart failure patients after an episode of acute decompensation: randomized controlled trial. *J Med Internet Res* 2009;**11**:e34
Schwarz KA, Mion LC, Hudock D, Litman G. Telemonitoring of heart failure patients and their caregivers: a pilot randomized controlled trial. *Prog Cardiovasc Nurs* 2008;**23**:18–26
Sisk JE, Hebert PL, Horowitz CR, McLaughlin MA, Wang JJ, Chassin MR. Effects of nurse management on the quality of heart failure care in minority communities: a randomized trial. *Ann Intern Med* 2006;**145**:273–283
Smith B, Hughes-Cromwick PF, Forkner E, Galbreath AD. Cost-effectiveness of telephonic disease management in heart failure. *Am J Manag Care* 2008;**14**:106–115
Soran OZ, Piña IL, Lamas GA, *et al.* A randomized clinical trial of the clinical effects of enhanced heart failure monitoring using a computer-based telephonic monitoring system in older minorities and women. *J Card Fail* 2008;**14**:711–717
Spaeder J, Najjar SS, Gerstenblith G, *et al.* Rapid titration of carvedilol in patients with congestive heart failure: a randomized trial of automated telemedicine versus frequent outpatient clinic visits. *Am Heart J* 2006;**151**:844.e1–10
Tompkins C, Orwat J. A randomized trial of telemonitoring heart failure patients. *J Healthc Manag* 2010;**55**:312–22
Tsuyuki RT, Fradette M, Johnson JA, *et al.* A multicenter disease management program for hospitalized patients with heart failure. *J Card Fail* 2004;**10**:473–480
Wade MJ, Desai AS, Spettell CM, *et al.* Telemonitoring with case management for seniors with heart failure. *Am J Manag Care* 2011;**17**:e71–e79
Wakefield BJ, Holman JE, Ray A, *et al.* Outcomes of a home telehealth intervention for patients with heart failure. *J Telemed Telecare* 2009;**15**:46–50
Wakefield BJ, Ward MM, Holman JE, *et al.* Evaluation of home telehealth following hospitalization for heart failure: a randomized trial. *Telemed J E Health* 2008;**14**:753–761
Weintraub A, Gregory D, Patel AR, *et al.* A multicenter randomized controlled evaluation of automated home monitoring and telephonic disease management in patients recently hospitalized for congestive heart failure: the SPAN-CHF II trial. *J Card Fail* 2010;**16**:285–292
Woodend AK, Sherrard H, Fraser M, Stuewe L, Cheung T, Struthers C. Telehome monitoring in patients with cardiac disease who are at high risk of readmission. *Heart Lung* 2008;**37**:36–45
Wootton R, Gramotnev H, Hailey D. A randomized controlled trial of telephone-supported care coordination in patients with congestive heart failure. *J Telemed Telecare* 2009;**15**:182–186

Table 7 Hypertension RCTs

Study	No of subjects	Type of pt	Intervention	Telemonitoring?	Duration (months)	Outcomes	Result	Overall value of intervention (on HbA1c)
Artinian 2007	387	≥140/90	BP readings (3 per week) sent via PSTN; nurses phoned back	Y	12	BP	Sig reduction in sBP relative to control group; NS reduction in dBP	MILDLY POSITIVE
Artinian 2001	26	≥140/90	BP readings (3 per week) sent via PSTN. Nurse counselling	Y	3	BP	Sig reduction in sBP and dBP relative to control group	POSITIVE
Bosworth 2009	636	Hypertension for at least 1 y [average was 125/71]	Four arm trial: 1. usual care; 2. telephone calls for self-management; 3. home BP monitoring; 4. both. In 3&4, pts took BP 3 times a week, recorded the values and posted the logs every 2 months	Y	24	Adequate BP control; Healthcare utilization; Cost	Sig increase in number of patients with controlled BP in combined group (4) compared to controls; NS diffs in utilization	POSITIVE
Brennan 2010	638 (485 completed follow-up)	Hypertension	Monthly phone calls from hypertension nurse to provide education and support. Self-monitored BP collected at 6 and 12 months	Y (sort of)	12	BP; Frequency of monitoring; Adherence; Resource utilization	Sig reduction in sBP relative to control group; Sig higher frequency of BP monitoring; NS diff in adherence or resource utilization	MILDLY POSITIVE (i.e. changes in 2 of about 6 outcomes)
Carrasco 2008	285	≥140/90	SMS transmission of BP, PR and weight four times/week	Y	6	BP; QoL	NS differences	NONE
Datta 2010	588	Hypertension	Nurse telephone call every 2 months	N	24	Resource utilization	NS difference in costs	NONE
Friedman 1996	267	≥160/90	IVR. Self-reported BP, knowledge etc	Y	6	Adherence; BP	Sig improvement in adherence; Sig reduction in dBP	POSITIVE
Green 2008a	778	≥140/90	Two interventions: Gp 1-web site; Gp 2-website plus pharmacist care management by phone/email	Unclear	12	BP	Sig improvement in BP in Gp 2	POSITIVE
Istepanian 2009	137	>130/80	Mobile network (3G) transmission of weekly BP readings to web site for doctor and patient	Y	9	BP	Sig fall in sBP in T/M group compared to control	POSITIVE
Madsen 2008a, 2008b, 2011	223	>150/95	BP readings (3 per week) sent via PDA/mobile network. Web site for patient and doctor access to readings	Y	6	BP; QoL; Cost	NS diff in BP between the two groups; Sig better QoL in T/M group relative to controls; T/M more costly (though more effective)	MILDLY POSITIVE
Parati 2009	329	>140/90	Home BP transmitted via modem to monitoring centre	Y	6	BP; Cost	Sig more T/M pts normalized their daytime BP than controls; No diff in costs	MILDLY POSITIVE
Rinfret 2009	223	≥130/80	BP and adherence data collected by IVR system every week	Y	12	24-h sBP and dBP	Sig better 24-h BP averages in T/M group compared to controls	POSITIVE
Rogers 2001	121	≥130/85	BP readings (3 per week) sent automatically via PSTN. Report sent to patient and doctor	Y	c3	BP	Sig reduction in sBP and dBP relative to control group	POSITIVE
Varis 2010	189	>140/90	Home BP measurement 3 times/week, with results sent to doctor by **post** (!) every 5 weeks	Y (sort of)	12	BP	Sig worsening in BP in T/M group compared to controls	NEGATIVE

Hypertension RCTs
Artinian NT, Flack JM, Nordstrom CK, *et al.* Effects of nurse-managed telemonitoring on blood pressure at 12-month follow-up among urban African Americans. *Nurs Res* 2007;**56**:312–322
Artinian NT, Washington OG, Templin TN. Effects of home telemonitoring and community-based monitoring on blood pressure control in urban African Americans: a pilot study. *Heart Lung* 2001;**30**:191–199
Bosworth HB, Olsen MK, Grubber JM, *et al.* Two self-management interventions to improve hypertension control: a randomized trial. *Ann Intern Med* 2009;**151**:687–695
Brennan T, Spettell C, Villagra V, *et al.* Disease management to promote blood pressure control among African Americans. *Popul Health Manag* 2010;**13**:65–72
Carrasco MP, Salvador CH, Sagredo PG, *et al.* Impact of patient-general practitioner short-messages-based interaction on the control of hypertension in a follow-up service for low-to-medium risk hypertensive patients: a randomized controlled trial. *IEEE Trans Inf Technol Biomed* 2008;**12**:780–791
Datta SK, Oddone EZ, Olsen MK, *et al.* Economic analysis of a tailored behavioral intervention to improve blood pressure control for primary care patients. *Am Heart J* 2010;**160**:257–263
Friedman RH, Kazis LE, Jette A, *et al.* A telecommunications system for monitoring and counseling patients with hypertension. Impact on medication adherence and blood pressure control. *Am J Hypertens* 1996;**9**:285–292
Green BB, Cook AJ, Ralston JD, *et al.* Effectiveness of home blood pressure monitoring, Web communication, and pharmacist care on hypertension control: a randomized controlled trial. *JAMA* 2008;**299**:2857–2867
Istepanian RSH, Sungoor A, Earle KA. Technical and compliance considerations for mobile health self-monitoring of glucose and blood pressure for patients with diabetes. *Conf Proc IEEE Eng Med Biol Soc* 2009;**2009**:5130–5133

Madsen LB, Christiansen T, Kirkegaard P, Pedersen EB. Economic evaluation of home blood pressure telemonitoring: a randomized controlled trial. *Blood Press* 2011;**20**:117–125
Madsen LB, Kirkegaard P, Pedersen EB. Health-related quality of life (SF-36) during telemonitoring of home blood pressure in hypertensive patients: a randomized, controlled study. *Blood Press* 2008;**17**:227–232
Madsen LB, Kirkegaard P, Pedersen EB. Blood pressure control during telemonitoring of home blood pressure. A randomized controlled trial during 6 months. *Blood Press* 2008;**17**:78–86
Parati G, Omboni S, Albini F, *et al.* Home blood pressure telemonitoring improves hypertension control in general practice. The TeleBPCare study. *J Hypertens* 2009;**27**:198–203
Rinfret S, Lussier M-T, Peirce A, *et al.* The impact of a multidisciplinary information technology-supported program on blood pressure control in primary care. *Circ Cardiovasc Qual Outcomes* 2009;**2**:170–177
Rogers MA, Small D, Buchan DA, *et al.* Home monitoring service improves mean arterial pressure in patients with essential hypertension. A randomized, controlled trial. *Ann Intern Med* 2001;**134**:1024–1032
Varis J, Kantola I. The choice of home blood pressure result reporting method is essential: Results mailed to physicians did not improve hypertension control compared with ordinary office-based blood pressure treatment. *Blood Press* 2010;**19**:319–324
